# Juvenile growth, thermotolerance and gut histomorphology of broiler chickens fed *Curcuma longa* under hot-humid environments

**DOI:** 10.1016/j.heliyon.2023.e13060

**Published:** 2023-01-16

**Authors:** C.C. Kpomasse, O.M. Oso, K.O. Lawal, O.E. Oke

**Affiliations:** a Centre d’Excellence Regional sur les Sciences Aviaires (CERSA), University of Lome, Togo; b Department of Animal Physiology, Federal University of Agriculture, Abeokuta, Nigeria

**Keywords:** Thermotolerance, Morphology, Juvenile growth, Broiler

## Abstract

**Background:**

This study was conducted to assess the juvenile development, thermotolerance, and intestinal morphology of broiler chickens fed Curcuma longa in a hot-humid environment.

**Methods:**

In a Completely Randomized Design, 240 broiler chicks were randomly assigned to four nutritional treatments of baseline diets supplemented with 0 (CN), 4 (FG), 8 (EG), and 12 g (TT) of turmeric powder/Kg of feed, with four replicates of fifteen birds each. Data on feed consumption and body weights were evaluated weekly during the juvenile growth phase. The physiological indicators of the birds were assessed on day 56 of their lives. The birds were subjected to thermal challenge and data were collected on their physiological traits. Eight birds were randomly selected, euthanized and dissected in each treatment, and 2 cm segments of duodenum, jejunum, and ileum were sampled for villi width, villi height, crypt depth, and the villi height: crypt depth ratio measurements.

**Results:**

It was revealed that the weight gain of the birds in EG was significantly greater (p < 0.05) than that of CN birds. The birds in TT, FG, and CN had comparable but smaller duodenal villi than those in EG. The ileal crypt depth in EG chickens was smaller than in CN but comparable to the other treatment groups. In the duodenum, the villi to crypt depth ratio was in the order EG > TT > FG > CN.

**Conclusions:**

To conclude, dietary supplementation of Curcuma longa powder, notably the 8 g/kg diet improved the antioxidant status, thermotolerance, and nutrient absorption by improving intestinal morphology in broiler chickens in a hot-humid environment.

## 1 Introduction

The adverse effect of thermal stress on livestock has been a growing global concern [[1], [2], [3], [4], [5], [6], [7], [8]]. Thermal stress occurs when the net quantity of energy moving from the animal's body to its surroundings and the amount of heat energy generated by the animal are out of balance [9] due to fluctuations in the weather elements. The thermoregulatory mechanism of birds is overstretched by thermal stress, resulting in changes in biological functions [10]. Broiler chickens perform optimally at the thermo-neutral zone of about 20–22 °C. However, tropical environments where the ambient temperature can be as high as 36 °C are counterproductive for chickens, particularly broiler chickens. The tropical environmental conditions, especially high ambient temperature and humidity, adversely influence chickens [11]. Previous findings have revealed that birds in hot-humid environments would make some physiological changes to survive at the expense of production [12,13], resulting in poor growth and high mortality. Moreover, thermal stress adversely impacts immunity [14].

Studies have shown that some morphologic changes are observed in the gastrointestinal tract integrity [15,16], including deterioration in intestinal morphological traits, such as villi height, crypt depth, villi width, the ratio between villi height and crypt depth under hot environment [17,18]. Indeed [19], stated that the gastrointestinal tract is predominantly responsive to heat stress.

Various methods of ameliorating the effect of thermal stress on chickens, including roof sprinklers and evaporative cooling systems [20] are not affordable for farmers in developing countries. Therefore, it is essential to search for possible ways of enhancing the thermotolerance of birds in hot climates to optimize their performance [21] by correcting the altered antioxidant status during heat stress [22]. Diets have been used to supply the altered nutritional requirement of stressed chickens. It has been reported that antioxidants reduce chemical radicals and disrupt lipid peroxidation to protect the cells from the effects of reactive oxygen species [23]. Moreover, there has been a growing interest in the use of phenolic compounds such as Curcuma longa as a consequence of their putative health effects with respect to their antioxidant, anticarcinogenic, anti-inflammatory, and antimicrobial activities [24].

Curcumin has been reported to be the main phenolic compound Curcuma longa powder with an antioxidant effect [4,25,26]. In addition to its antioxidant properties, the free radical scavenging properties [27], hypolipidemic effects [28], protection of biological membranes from peroxidative damage [29], enhancement of immune function [30], and antiviral and antibacterial properties [31] of *Curcuma longa* have been reported. The antioxidant suppresses lipid peroxidation [32] but increases detoxifying enzyme actions [33]. Earlier studies have also identified biological activities such as the anticoagulant [34], improvement of the nutrients digestibility and metabolism [35], hepatic functions [36], reduction of serum LDL, cholesterol, triglycerides, and blood glucose [26]. Recent studies have shown that curcumin in Curcuma longa improved the performance of broiler chickens under thermal stress conditions [4,23].

Despite the plethora of information on the evaluation of dietary Curcuma longa on the performance of broilers [4,[37], [38], [39], [40]]; the mechanism of action of this phytogenic feed additive, however, is not completely elucidated. Data are also limited on the effects of *Curcuma longa* on the amelioration of heat stress in broilers under hot-humid environments. Therefore, this study aimed to evaluate the thermotolerance, intestinal morphology, and juvenile growth performance of broiler chickens fed Curcuma longa under hot-humid environments.

## 2 Materials and methods

### 2.1 Ethical permit

The Animal Experimental Board of the Department of Animal Physiology, College of Animal Science and Livestock Production, Federal University of Agriculture, Abeokuta, Nigeria, authorized the experiment, and the Nigeria Institute of Animal Science (NIAS) standard for Animal Research was followed. Animal cruelty and unnecessary suffering were avoided.

### 2.2 Chickens, diets and management

Dried *Curcuma longa* rhizomes powder was obtained from a renowned local spices store. The following dietary treatments were tested: CN (Corn-soy based basal diet with no *Curcuma longa* powder), FG (Corn-soy based basal diet supplemented with 4 g per kilogram), EG (Corn-soy based basal diet supplemented with 8 g per kilogram), and TT (Corn-soy based basal diet supplemented with 12 g per kilogram) diets. The treatment was applied from a day old and lasted for 56 days.

A total of 240 day-old chick broilers (Marshal) were sourced from a reliable hatchery. The birds were randomly assigned to four dietary treatments having four replicates each, with fifteen birds. Each of the four pens holding 15 chickens (n = 15) in each treatment was used as an experimental unit. The chicks were reared using a conventional commercial management practice. The chickens were reared on a deep litter (wood shavings) floor in an open-sided poultry house. Ad libitum feeding was adopted throughout the experiment and the diets were supplied to match NRC (1994) nutritional guidelines. Throughout the experiment, water at room temperature was available at all times. The average meteorological data of 31.6 °C and 77.75% of temperature and relative humidity, respectively, was recorded during the study. The experiment lasted for 56 days.

### 2.3 Data collection

#### 2.3.1 Juvenile growth

The growth performance of the birds was determined using the method of [90].

#### 2.3.2 Body weight gain

The weights (in gram) of the chicks were taken on a replicate basis using a weighing scale. The weight gain was determined as the difference in the weights of the birds weekly.

#### 2.3.3 Feed intake

The weight of the leftover feed was subtracted from the weights of feed supplied to get the amount of feed consumed by the birds.

#### 2.3.4 Feed conversion ratio

The ratio of feed intake and weight gain was used to determine the feed conversion ratio (FCR) of the birds using the formula below:

(1) FCR = Total feed consumed _(g)_/Body Weight gain _(g)_

The experimental unit for the growth performance was 60 birds.

### 2.4 Blood parameters

Blood samples were collected from 8 birds per treatment (2 birds per replicate) for the determination of pH and haematological parameters. The samples were collected from the brachial vein into heparinized tubes and the parameters were determined following the methods of [41].

About 2 ml blood samples were obtained from the brachial veins of 8 birds per treatment after they were thermally challenged. The samples were centrifuged for 15 min (1800 ×*g*) and the plasma obtained was used for malondialdehyde, uric acid and triiodothyronine determination. Plasma triiodothyronine was analyzed with the use of commercial kits and an ELISA reader. Plasma malondialdehyde (MDA) was determined with the use of colourimetric methods with a spectrophotometer. The description of [42] was followed using commercial kits. The determination of plasma uric acid was done using available commercial kits (Sigma Diagnostics, Missouri).

### 2.5 Intestinal morphology

On day 56, the chickens were deprived of feed overnight; eight chickens were then randomly selected per treatment (2 per replicate), weighed, and euthanized. The birds were dissected and samples (8 samples per treatment) (2 cm long) were taken from the ileum, jejunum and duodenum. The samples were flushed with physiological saline to remove intestinal contents and placed in a labelled bottle containing 10% formalin for morphological measurement. The method of [24] was used for tissue processing and staining. The tissues were examined under the microscope for the morphometric analysis of and crypt depth, villus height, villi width and villus height:crypt depth ratio [43]. Each intestinal cross-section was used to calculate the depth of the crypt and the heights of 8 intact, well-oriented villi, as described by Ref. [44]. Villus width was taken as the villus' midline, and villus heights were calculated as the tipoff of the villus crypt junction. The muscularies layer to the serosa was used to estimate the mucosa wall thickness.

### 2.6 Physiological measurements

This was determined between 1 and 2 p.m. in week 6. The number of breaths per minute was used to calculate the birds' respiratory rate. A stethoscope was gently placed on the chest region of the birds to take the reading within 15 s. The measurements were multiplied by four to get the values per minute. The rectal temperature was determined by gently inserting a digital thermometer into the bird's rectum and the reading was taken when a beep sound was made. The skin temperature on the comb was measured using an infrared thermometer.

### 2.7 Statistical analysis

Data obtained in this study were subjected to analysis of variance for a Completely Randomized Design using [45] statistical package and significant means were compared using Tukey's HSD test. GraphPad Prism 5.0 was used in creating the graphics. The difference was considered significant when P < 0.05.

## 3 Results

The juvenile growth performance of birds supplemented with dietary *Curcuma longa* in a hot-humid environment is shown in Table 1. The weights of the chicks offered *Curcuma longa* were similar but significantly higher (P < 0.05) than that of the CT treatment group at week 2. The increase in the weight of the chicks in TT and FG was similar to that of CT; however, the weight gain of the chicks in EG was significantly higher. The birds in each treatment group had a similar feed intake. The FCR of the CT birds was not different from those of the FG and TT birds but greater than that of the EG birds.

**Table 1:** The juvenile growth performance of birds supplemented dietary *Curcuma longa* in a hot-humid environment.

Trait	CN	FG	EG	TT	SEM	P Value
Initial weight (g) (day old)	41.09	41.92	41.08	41.86	0.168	0.1216
Final weight (g) (2 weeks)	226.08^b^	227.76^a^	228.26^a^	227.46^a^	0.212	0.0004
Weight gain (g)	184.99^b^	185.85^ab^	187.18^a^	185.60^ab^	0.258	0.0145
Feed intake (g)	297.82	290.65	282.87	284.66	2.401	0.1087
FCR	1.61^a^	1.56^ab^	1.51^b^	1.53^ab^	0.013	0.0390

^a,b:^ The means of different superscripts in the same row varied significantly (P < 0.05), 0 (CN), 4 (FG), 8 (EG), and 12 g (TT)/Kg diet.

**Fig. 1:**
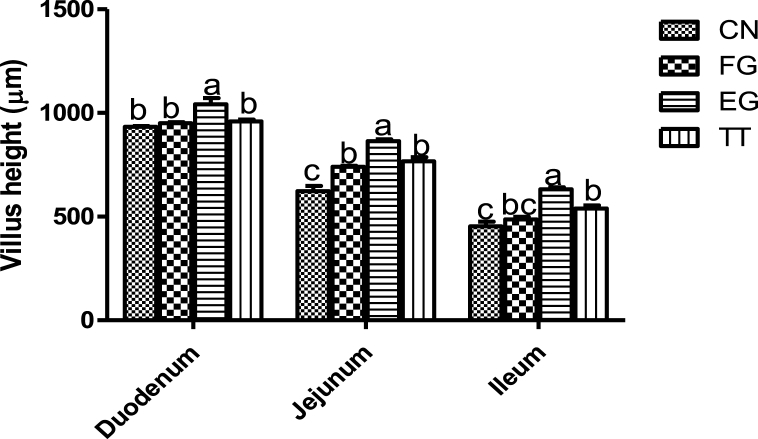
Villus heights chickens supplemented dietary *Curcuma longa under hot-humid environments.*

The villus height of birds fed *Curcuma longa* is shown in Fig. 1. The duodenal villi height of the birds in EG was higher than in other treatments. Ileal heights of the birds in TT were similar to that of FG but significantly higher than EG. Moreover, the jejunal height of the birds in EG was higher than those of the other treatment groups, while those of TT and FG were similar but higher than those of CN.

**Fig. 2:**
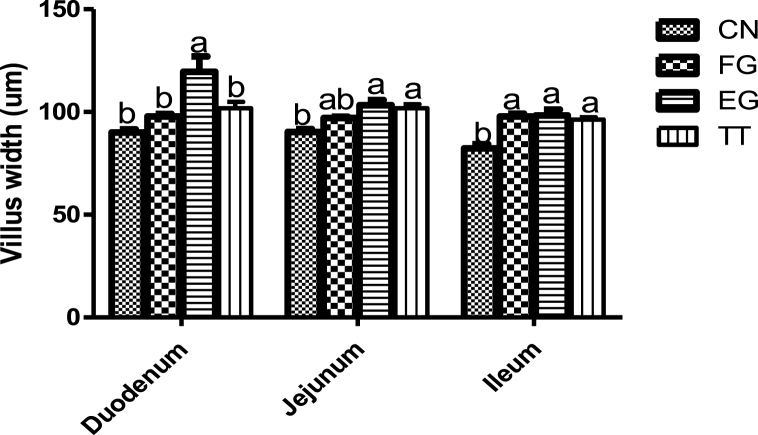
Villus width of broiler chickens supplemented with different levels of *Curcuma longa.*

The villi width of birds supplemented with *Curcuma longa* is presented in Fig. 2. The duodenal villi width of the birds in TT, FG and CN was similar but lower than that of EG. The ileal villi width of the chickens of the control was significantly lower than those of the birds fed different levels of *Curcuma longa,* whose values were similar. Jejunal villi widths in EG and TT were similar but higher than that of CN.

**Fig. 3:**
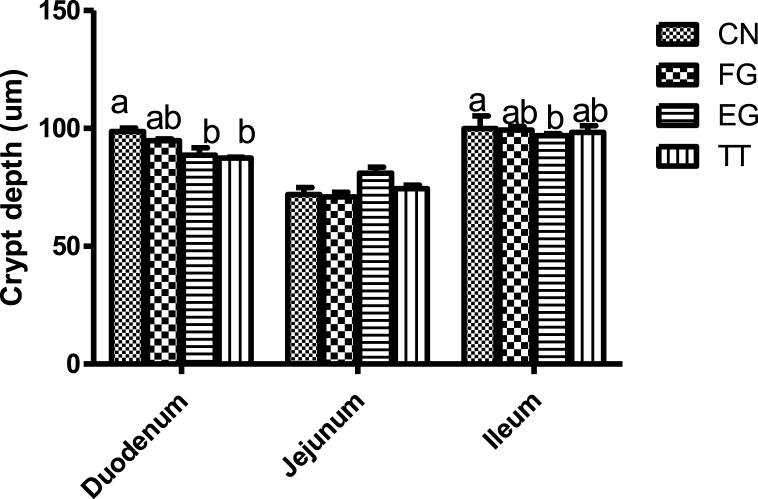
Crypt depth of broiler chickens supplemented with *Curcuma longa.*

The crypt depth of birds supplemented with *Curcuma longa* is presented in Fig. 3. Duodenal crypt depth of the birds in CN was similar to that of FG but higher than EG and TT. The crypt depth of those in FG was similar to EG and TT. Chickens in EG had a lower ileal crypt depth than those in CN, but they were similar to those in the other treatment groups.

**Fig. 4:**
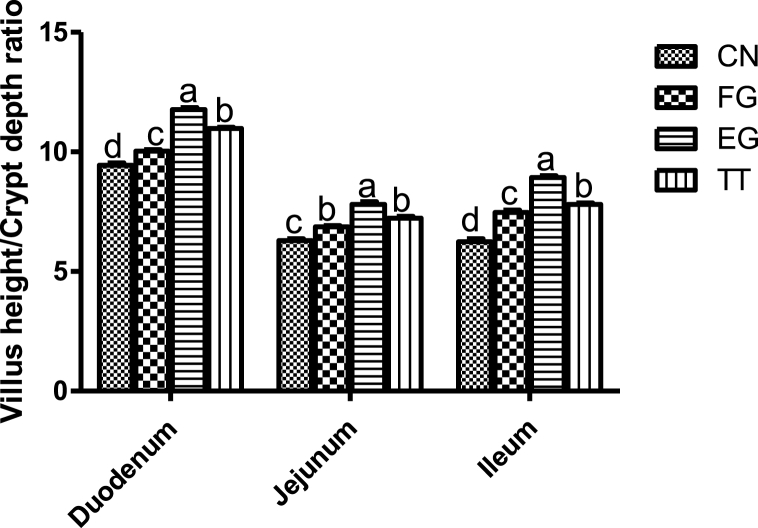
Villus height/crypt depth ratio of broiler chickens supplemented with *Curcuma longa.*

In the duodenum of the chickens, the ratio of villi height to crypt depth was in the order EG > TT > FG > CN. (Fig. 4). In the ileum, the ratio was greater in EG than FG, which was higher than TT and higher than CN. However, in the jejunum, FG and EG were similar and higher than CN but lower than TT.

**Fig. 5:**
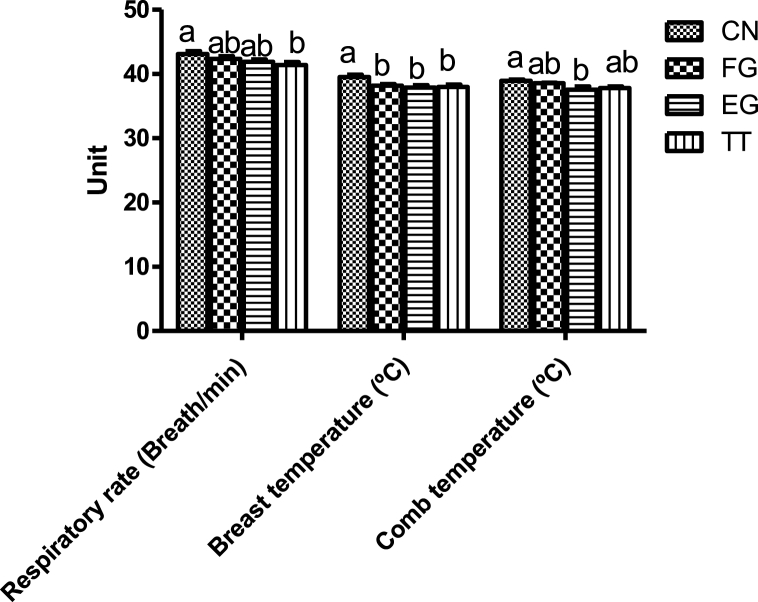
Respiratory rate and breast and comb temperatures of broiler chickens supplemented with *Curcuma longa at market age.*

**Fig. 6:**
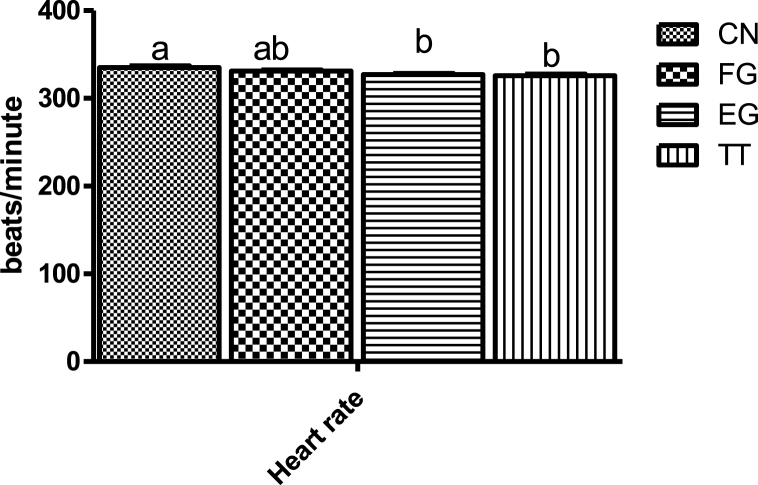
Heart rate of broiler chickens supplemented with *Curcuma longa* at market age.

The respiratory rate, comb and breast surface temperatures of broiler chickens fed *Curcuma longa* under humid tropical environments are shown in Fig. 5. The respiratory rate of the birds in FG and EG was intermediate between those of CN and TT. The respiratory rate of CN birds was significantly higher than those of TT. The breast surface temperatures of the birds of FG, EG and TT were comparable but lower than CN. The comb temperature of the CN chickens was significantly compared to EG birds but was not different from the others. The heart rate of CN birds was not significantly different from FG but higher than that of TT and EG birds (Fig. 6).

The haematological parameters of the birds fed *Curcuma longa* in a hot-humid condition are shown in Table 2. There was no difference in the chickens' PCV, haemoglobin, red blood cells, white blood cells, and lymphocyte across different treatment groups. However, the heterophil of the chickens in CN was significantly higher than those of the other treatments. Additionally, the heterophil/lymphocyte of the birds in CN was similar to FG birds but lower than EG and TT chickens. The heterophil/lymphocyte of FG chickens was comparable to those of TT but higher than the ratio of EG chickens.

**Table 2:** The haematological indices of broiler chicken fed *Curcuma longa* during hot-humid environment.

Treatment	CN	FG	EG	TT	SEM	P value
PCV %	30.50	37.75	42.00	34.75	2.211	0.3291
Hb(g/dl)	11.75	12.55	13.23	13.40	0.767	0.8939
RBC(×10^12^/l)	2.95	2.73	2.50	2.94	0.233	0.9088
WBC(×10^9/L^)	16.35	13.78	11.90	15.03	0.932	0.4102
Heterophil	36.75^a^	28.50^b^	26.75^b^	29.24^b^	1.244	0.0062
Lymphocyte	47.75	45.50	57.74	58.26	2.338	0.0927
H/L	0.78^a^	0.64^ab^	0.47^c^	0.51^bc^	0.036	0.0005

^a,b:^ The means of different superscripts in the same row varied significantly (P < 0.05), 0 (CN), 4 (FG), 8 (EG), and 12 g (TT)/Kg diet.

Table 3 shows the physiological responses of broiler chickens fed *Curcuma longa* thermally challenged at market age. The plasma uric acid of the birds was not affected by the treatments. However, the plasma MDA of EG birds was lower than those of FG and CN, which were comparable with those of TT birds. The rectal temperature of EG birds was not different from those of TT but lower than the other treatment groups. The plasma triiodothyronine of TT and FG birds was comparable and higher than those of CN birds. Additionally, the concentration was higher in EG birds than in other groups.

**Table 3:** Physiological responses of broiler chicken fed *Curcuma longa* thermally challenged at market age.

Parameter	CN	FG	EG	TT	SEM	P Value
MDA	0.78^a^	0.72^a^	0.67^b^	0.75^ab^	0.013	0.0277
Rectal temperature (C)	42.88^a^	42.43^b^	41.85^c^	42.14^bc^	0.079	0.0001
Triiodothyronine (ng/ml)	0.87^c^	1.33^b^	1.74^a^	1.40^b^	0.060	0.0001
Uric Acid (ng/ml)	11.34	13.08	13.68	13.47	0.548	0.4330

^a,b:^ The means of different superscripts in the same row varied significantly (P < 0.05), MDA: Malondialdehyde, 0 (CN), 4 (FG), 8 (EG), and 12 g (TT)/Kg diet.

## 4 Discussion

Thyroid hormones are biomarkers of different types of stress in chickens [46]. The findings in the present study demonstrate that the birds supplemented 8 g/kg diet *Curcuma longa* in this study had a higher concentration of triiodothyronine. This observation suggests that the bioactive compound of *Curcuma longa* at this dose had a positive effect on the birds during the thermal challenge. Additionally, the enhanced antioxidative status can be ascribed to the improved triiodothyronine as the activity of antioxidant enzymes is regulated by thyroid hormones [47]. The findings of [48] also demonstrated that the plasma concentrations of MDA were lowered by the circulating triiodothyronine hormone. The lower concentrations of triiodothyronine observed in the birds in the control group in the present study is consistent with the findings of [49], who demonstrated that acute heat stress depressed thyroid hormone [50]. indicated that the activity and size of the thyroid gland were reduced by heat stress. As an adaptive strategy, this mechanism decreases metabolic heat production, reduces maintenance energy requirements, and promotes fat deposition by discouraging lipolysis to escape extra heat load and ensure survivability [51,52]. The higher T3 observed in the birds fed *Curcuma longa* demonstrates that the impact of the thermal challenge was ameliorated by the bioactive compounds of the phytogenic feed additive.

An increased heart rate is one of birds’ homeostatic responses to stress [9]. Under heat stress, an increase in heart rate triggers catecholamine release (adrenaline and noradrenaline) in the adrenal glands [53]. Generally, the high heart rate of the broiler chickens in this study suggests that they were stressed and the lower heart rate recorded in the birds of EG and TT indicates that these doses of the doses were able to attenuate the effect of the harsh environment on the birds. In response to stress, the parabrachial nucleus is stimulated, thus increasing the rate of respiration [54]. The TT birds had a lower respiratory rate in this study, suggesting the positive effect of the additive. Generally, the respiratory rate of the birds appeared to be dose-dependent. Body temperature is an excellent measure of metabolic rate and is associated with acclimatization [55]. An increase in respiration rate in birds exposed to heat stress has been reported to decrease the blood HCO3- and PCO2 and increase blood pH, resulting in respiratory alkalosis [56]. However, there was no significant effect of dietary *Curcuma longa* on the blood pH of the present study. The elevated rectal temperatures of the birds subjected to acute heat stress in this study are in congruence with the existing literature [57,58], Haematological parameters reflect the health status of an animal. They are important biomarkers of physiological and nutritional status [59]. Most of the haematological parameters of the birds in the present study were not affected by the supplementation of *Curcuma longa*, indicating the safety of the phytogenic feed additive. In poultry, heterophil/lymphocyte has often been used as an indicator of stress [60]. Thermal stress has been shown to increase H/L ratios [61,62]. The lower heterophil/lymphocyte of EG and TT chickens in this study indicates that they were less stressed than the birds in the control groups. This implies that the bioactive compounds of *Curcuma longa* were beneficial in regulating the thermotolerance of the birds. The acid-base balance of chickens can be affected by the environmental temperature [58].

The small intestine is an important organ for the absorption of nutrients, and it can also serve as a growth mechanism indicator [63]. The mucosa structure of the intestine can reflect the health condition of the gut and gut health can be manipulated to enhance feed efficiency and growth performance [24,64]. The villi height and width of the birds offered dietary *Curcuma longa* were improved in the present study, particularly at the EG birds' duodenum, jejunum, and ileum. This observation corroborates the findings of [40,65], who demonstrated that curcumin and turmeric powder supplementation enhanced the intestinal morphology of broiler chickens. Similarly, the findings of [42] revealed that dietary turmeric improved the villi heights of the duodenum. As the small intestine epithelial villi become longer because of mitotic division, they broaden the intestine area, increasing nutrient absorption [42]. The absorption capability of animals with damaged or short intestinal villi is impaired due to a smaller absorption area of the intestine, leading to poor feed efficiency and growth [66]; however, in the small intestine, longer villi would provide more absorption area for nutrients, which could improve nutrient absorption [66,67]. *Curcuma longa* exerts its effect on gut health by lowering the bacterial load and the pH of the intestine while selectively increasing beneficial bacteria (Lactobacillus) [68]. The improvement in the intestinal morphology of the birds under a hot-humid environment in this trial indicates that *Curcuma longa* could ameliorate the effects of stress conditions [69]. have demonstrated that intestinal morphology is impaired by stress. The higher villus height:crypt depth ratio recorded at the different segments of the intestine in the present study suggests better nutrient absorption. This may explain the weight gain of the birds in these treatment groups. The crypt depth of the birds fed 8 g/kg diet was shallower at the duodenum and ileum segments of the intestine in the present study, indicating that the additive was beneficial to the gut health of the birds at this dose. Nutrient absorption and growth performance can be compromised by deeper crypts [70]. A large crypt indicates rapid tissue turnover and high demand for new tissue, as the crypt can be considered a villus factory [64]. The presence of toxins or higher tissue turnover has been associated with deeper crypts [71,72].

As an indicator of lipid peroxidation, MDA is widely used as a biomarker of stress and since its levels change in response to heat stress, it is considered a reliable marker when birds are exposed to a thermal challenge [73,74]. The decrease in the MDA of birds of EG compared to the control birds in the present study suggests that curcumin, the bioactive compound in the *Curcuma longa*, was beneficial to the birds in attenuating the adverse effect of the thermal challenge to the birds were subjected. This observation is in harmony with the observation of [75], who demonstrated that chicks from the hatching eggs treated with curcumin had improved MDA levels. Additionally [76,77], reported that oxidant damage induced by thermal challenge was attenuated by curcumin. Similarly [78], indicated that the cells’ antioxidant status was maintained with the use of dietary curcumin in rats. Indeed [79], demonstrated that curcumin's phenolic structure could trap free radicals and produce stable and strong anthraquinones. Curcumin is thought to have anti-inflammatory properties through scavenging free radicals and activating antioxidant enzymes and hepato-protective effects [78,80].

Reduced synthesis and secretion of triiodothyronine and increased conversion of thyroxine are two cellular mechanisms that animals use to lower heat production as the ambient temperature rises [81]. The lower plasma triiodothyronine of the birds in the control in the present study corroborates earlier studies indicating that birds subjected to heat stress [82,83]. However, the higher plasma triiodothyronine recorded in the birds fed *Curcuma longa* in this present study depicts a better thermotolerance by the birds, suggesting the beneficial effect of the bioactive compounds of *Curcuma longa*. Consistent with our result [75], demonstrated chicks from hatching eggs sprayed with curcumin had higher plasma triiodothyronine.

Chickens in their thermoneutral zone, being endothermic animals, can maintain a core body temperature of 41 °C through latent and sensible thermal losses [84]. However, when they are exposed to thermal challenges, they are unable to maintain a balance between body heat generation and heat removal, resulting in increased skin temperature and, as a result, a rise in core body temperature, which can lead to mortality [9]. Generally, the rectal temperature of the birds thermally challenged was elevated. However, the lower rectal temperature in the birds fed *Curcuma longa* suggests the birds had inherent higher tolerance to withstand heat stress.

The juvenile growth of broilers is linearly correlated to the bodyweight at market age [85–87]. Bodyweight at an early age appeared to be the best predictor of BW at slaughter age [85,86]. The improved weight gain and feed conversion ratio of EG birds compared to the control group in the present study is consistent with the existing literature [88,89]. The enhanced performance can be explained by the chickens' improved gut health and antioxidant status in this study. The similarity in the feed intake in the present study is in agreement with the report of [76]. Consistent with our findings, the authors also reported that the feed conversion ratio of quail was enhanced by curcumin under heat stress.

## 5 Conclusion

Based on the findings obtained in this study, it could be concluded that supplementation of *Curcuma longa* ameliorated the effect of heat stress and *Curcuma longa* at 8 g/kg improved nutrient absorption and juvenile growth of broiler chickens through enhanced intestinal morphology under hot-humid tropical environment. It could be recommended that farmers in tropical environments could incorporate *Curcuma longa* in the diets of broiler chickens.

## Author contribution statement

Oyegunle Emmanuel Oke: Conceived and designed the experiments.

Oyegunle Emmanuel Oke, Claude Kpomasse, Oluwadamilola Oso, and Claude Kpomasse: Analyzed and interpreted the data; Performed the experiments; Contributed reagents, materials; Wrote the paper.

## Funding statement

This research did not receive any specific grant from funding agencies in the public, commercial, or not-for-profit sectors.

## Data availability statement

Data will be made available on request.

## Declaration of interest's statement

The authors declare no conflict of interest.
